# Laxative Properties of Microencapsulated Oleic Acid Delivered to the Distal Small Intestine in Patients with Constipation after Bariatric Surgery or Treatment with Glucagon-Like- Peptide 1 Analogues

**DOI:** 10.1007/s11695-024-07492-y

**Published:** 2024-09-05

**Authors:** Ahmed W. Al-Humadi, Werd Al-Najim, Sinead Bleiel, Carel W. le Roux

**Affiliations:** 1 Diabetes Complications Research Centre, Conway Institute, University College Dublin, Dublin, Ireland; 2 Diabetes Research Centre, Ulster University, Coleraine, BT52 1SA UK; 3 AnaBio Technologies Ltd, Carrigtwohill, Cork, T45 RW24 Ireland

**Keywords:** Bariatric surgery, Chronic constipation, GLP-1 analogues, Obesity, Oleic acid, Olive oil, Intentional weight-loss intervention

## Abstract

**Background:**

Constipation is prevalent after bariatric surgery and glucagon-like-peptide 1 (GLP-1) analogues. Increasing fat content in the distal small intestine and colon can enhance colonic peristalsis, potentially alleviating symptoms of constipation.

**Aim:**

We investigated whether oleic acid can ameliorate constipation in patients undergoing bariatric surgery or receiving GLP-1 analogues.

**Methodology:**

Fourteen adults with chronic constipation according to Rome IV criteria following bariatric surgery or GLP-1 analogues were on stable treatment for constipation for more than 4 weeks. This randomized double-blind crossover trial compared microcapsules containing 21.25 g of oleic acid delivered in the distal small intestine or the stomach. The primary outcome was changed in the number of bowel motions over 24 h. Exploratory endpoints included alterations in straining, diarrhoea, faecal leakage over 24 h and hunger, fullness, nausea and calorie intake for the 3 h after ingesting the microcapsules.

**Findings:**

Receiving oleic acid into the distal small intestine increased number of bowel movements per day (2.5 vs 1.1, *p* = 0.009) and caused softer stool consistency (*p* = 0.03). 9/14 of the control group passed motions and 13/14 of the intervention group passed motions in 24 h (*p* = 0.059). No significant differences were observed in straining (*p* = 0.65), rapid bowel movements (*p* = 0.08), accidental leakage (*p* = 0.32), hunger, fullness, nausea or food intake between the groups (all *p* > 0.05). There were no disparities in safety profile between groups.

**Conclusion:**

Microcapsules containing oleic acid delivered to the distal small intestine appear to be a safe and effective relief from chronic constipation in patients undergoing bariatric surgery and/or receiving GLP-1 analogues.

**Graphical Abstract:**

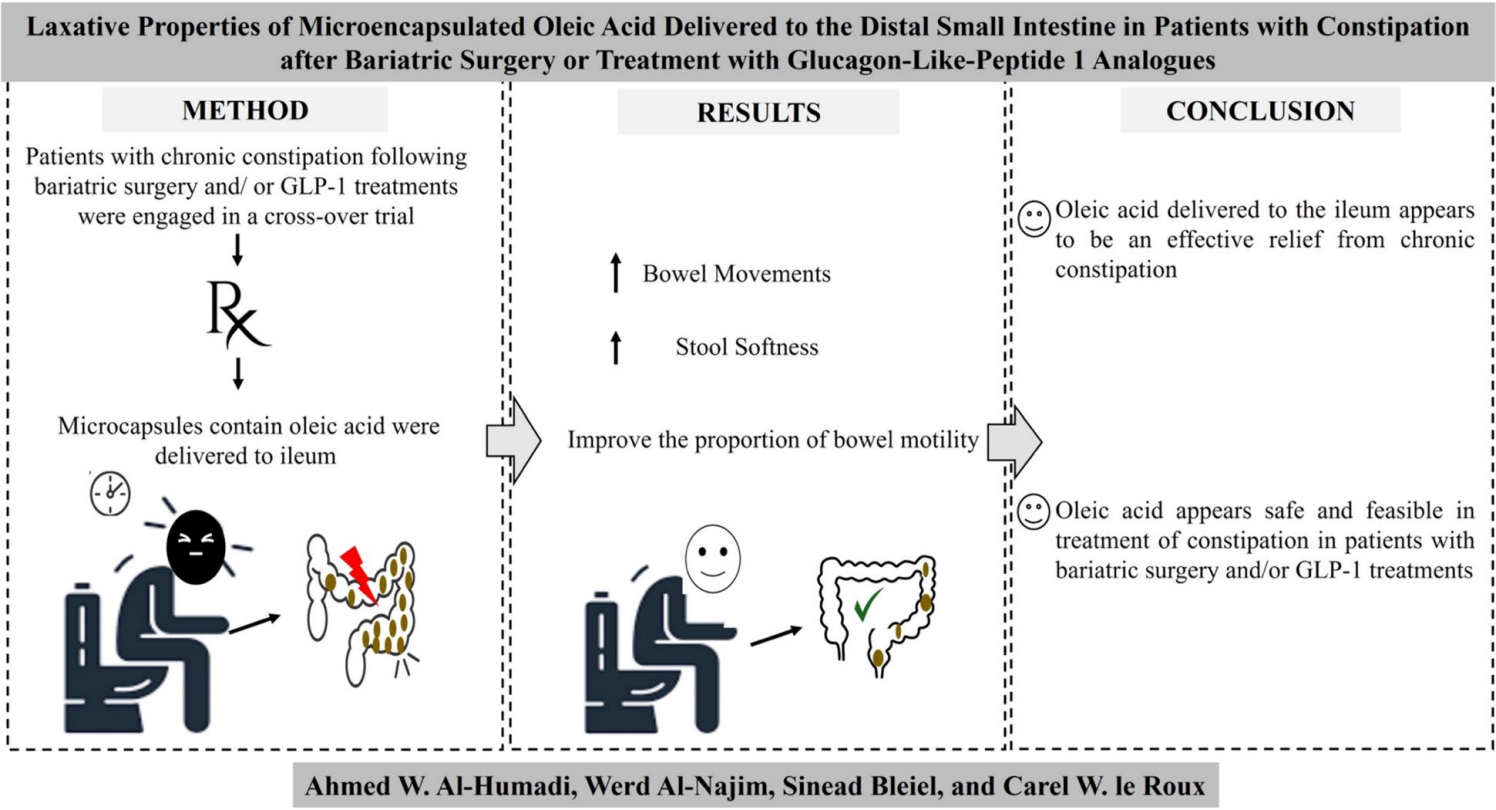

**Supplementary Information:**

The online version contains supplementary material available at 10.1007/s11695-024-07492-y.

## Introduction

Chronic constipation is a functional disorder affecting the regularity of bowel movements, persisting for a minimum of 3 months. The aetiology is multifactorial, involving dietary and lifestyle factors within the context of normal colonic motility. Confirmatory clinical diagnosis of chronic constipation is established using the Rome IV criteria [1]. Risk factors for constipation encompass various factors, including female sex, ageing, obesity, poor physical activity, low socioeconomic status, diabetes mellitus, medications and dietary factors [2–5]. Obesity and obesity treatments are associated to constipation; however, the association between obesity and constipation remains contentious [6, 7]. While laxatives are the most commonly prescribed treatment option, their long-term use is linked to potential side effects [8, 9].

Constipation frequently emerges as a significant side effect, for patients undergoing various treatments for obesity, including nutritional therapies [10], pharmacotherapies [11] or surgical interventions [12–14]. Notably, calorie reduction, a common strategy across these interventions, diminishes stimulation of colonic motility [15, 16], while intensive treatments for obesity decreases in dietary fibre during reduced food intake can lead to stool debulking. GLP-1 analogues and bariatric surgery may also independently reduce bowel motility [17–19].

Various dietary and pharmacological strategies have been explored to alleviate constipation during weight loss. Nonetheless, compliance with these approaches poses a challenge due to gastrointestinal side effects [7, 20, 21].

In our previous study, we demonstrated that a food encapsulation technology employing natural food-grade pea protein to deliver 500 kcal pure oleic acid (a polyunsaturated fatty acid abundant in olive oil) to the distal small intestine resulted in both attenuation of food intake and enhancement of enteroendocrine satiety hormone release [22] Importantly, we observed an increase in the frequency of bowel movements over the subsequent 24 h in half of the participants [22].

The potential laxative properties of orlistat, a pancreatic lipase inhibitor used in obesity treatment, have been investigated for idiopathic chronic constipation and constipation associated with opioid pain medication and antipsychotic (clozapine) therapy [23–25]. The mechanism underlying this improvement may involve the prokinetic effects of undigested fat to the colon.

Based on our preliminary findings, the current study sought to assess the potential utility of encapsulated fat, specifically oleic acid, delivered to the distal small intestine in alleviating chronic constipation among patients undergoing treatment with GLP-1 analogues or bariatric surgery.

## Material and Methods

### Study Design

The study was conducted with approval from St. Vincent’s University Hospital Research and Ethics Committee (protocol No. RS21-058). Fourteen patients receiving treatment for obesity and experiencing chronic constipation, as per Rome IV criteria [1] provided written informed consent to take part in the study from May 2023 to December 2023, and it was registered in ClinicalTrials.gov PRS Registry under study registration numbers: NCT05324241.

Inclusion criteria comprised patients aged 18–65 years with obesity, actively engaged in obesity treatment, diagnosed with chronic constipation (Rome IV criteria) and on stable treatment of constipation for at least 4 weeks before study commencement. Randomization software (https://www.randomizer.org) allocated participants to either a 250-ml drink with microcapsules that opened in the stomach containing 21.25 g of oleic acid (400 kcal), or microcapsules that opened in the distal small intestine containing 21.25 g (400 kcal) of oleic acid, provided by AnaBio® Technologies. The encapsulation technology used natural pea protein microcapsules. The randomization process was performed by an independent researcher. Concealed assignment was used, and the codes were only revealed at the study’s conclusion.

Participants attended two separate visits at weekly intervals after fasting for at least 14 h. A standard 100-mm visual analogue scale (VAS) questionnaire was administered to assess hunger, nausea and meal palatability before the drink, every 30 min over 3 h, and after meal consumption. Participants consumed one of the two randomised drinks containing microcapsules with 400 kcal of oleic acid or placebo (empty microcapsules). They were instructed not to alter their ongoing medical treatment, especially for constipation, throughout the study.

After consuming the drink there was a 3-h observation period. Participants were then offered a standardized ad libitum gluten-free Tesco meal (450 g per serving), including options such as chicken korma and rice (475 kcal) or tikka masala and rice (468 kcal), according to their preference (see Supplementary file 1). The meal was heated in the microwave for 3 min at a moderate-high temperature before serving. Participants were allotted 20 min in private to consume the lunch, after which their calorie intake was recorded. Participants were then discharged home and provided with a 24-h gastrointestinal diary to report bowel movements, symptoms and any adverse events occurring up to 24 h post-study.

### Microcapsule Design

Microcapsules were produced in accordance with Good Manufacturing Process standards (AnaBio Technologies, Carrigtwohill, Cork, Ireland). Proteinaceous microcapsular material was denatured and subsequently used to separately microencapsulate specific for oleic acid for (a) distal small intestine release and (b) stomach release. Microencapsulation was achieved by mixing the denatured proteinaceous capsular material and the active ingredient (oleic acid), followed by coextrusion technique. The finished material to be used in the subsequent trial was in a dry, flowable powder format. The recommended microcapsule production process temperature was 20 to 25 °C, because higher temperatures, especially in combination with turbulence, can lead to reduced microencapsulation efficiency and losses of active material. Zeta potential was used to determine both attractive and repulsive features between the active agent and (matrix) proteinaceous material within the microcapsules generated. The magnitude of interactions was optimized at 625 mV to ensure the generation of microcapsules with electrostatic potential most suitable for storage in a dry format without oxidative, moisture or acidic stress.

### Statistical Analysis

Statistical analysis was performed for the endpoints of the present study. The primary endpoint was the number of participants reporting completed bowel motion within 24 h. Exploratory endpoints included the number of bowel motion in 24 h, nausea, accidental leakage, straining and stool consistency (evaluated using the Bristol Stool Scale) within 24 h [26]. Additionally, appetite scores measured by visual analogue scale (VAS) and the number of consumed calories at the end of visits were assessed.

Student-paired two-tailed *t* test, McNemar, and two-way ANOVA repeated measures were used for comparison. The statistical analysis was performed using R software (version 4.2.3, 2022; https://www.R-project.org). The significance level for all analyses was set at *p* ≤ 0.05, 95% confidence interval. Data are expressed as *mean* ± *SEM*.

## Results

Table 1 lists the study population’s age, gender, ethnicity and obesity interventions.

**Table 1:** Demographics and characteristics of the study population

Characteristic	Value mean ± SEM (*N* = 14)
Age (years)	51.6 ± 11.0
Gender	
Female (%)	78.6% (11)
Male (%)	21.4% (3)
Height	166.9 ± 3.2
BMI (kg/m^2^)	35.9 ± 3.5
Ethnicity	
White Irish	92.9% (13)
Other (Asian)	7.1% (1)
Waist circumference (cm)	106.0 ± 5.8
Weight loss interventions	
Surgery	35.7% (5)
Sleeve gastrectomy	80% (4)
Roux-en-Y-bypass	20% (3)
Pharmacotherapy	35.7% (5)
GLP-1 agonist	100% (5)
Surgery and pharmacotherapy	28.6% (4)
Sleeve or Roux-en-Y plus GLP-1 agonist	
Laxative treatments for preceding 4 weeks	
Movicol, stimulant	57.1% (8)
Senokot, stimulant	14.3% (2)
Dulcolax, stimulant	7.1% (1)
Duphalac, osmotic	7.1% (1)
Combination	14.4% (2)

*BMI* body mass index, *cm* centimetre, *GLP-1* glucagon-like peptide receptor agonists, *kg* kilogram, *m* metre, *n* number, *SEM* standard error of the mean

### Characteristic of Gastrointestinal Symptoms

The group receiving oleic acid into the distal small intestine had 2.5 bowel movements per day compared with 1.1 bowel motions in the control group who received oleic acid into the stomach (*p* = 0.009). Nine of the fourteen participants in the control group and 13 of the 14 participants in the active treatment reported a completed bowel motion within 24 h (*p* = 0.059). Table 3 shows the active arm also has softer stool consistency (*p* = 0.03). Additionally, during the first bowel movement, the stool was significantly softer (*p* = 0.03) following consumption of oleic acid microcapsules in the distal small intestine vs stomach (Table 3). Table 3 also shows there are no statistically significant differences in straining (*p* = 0.65), rapid bowel movements (*p* = 0.083) or accidental leakage (*p* = 0.32) between the two groups. Before meal initiation, no differences were observed in hunger, fullness and nausea between the groups (Table 2). Moreover, the number of calories consumed during the meal was similar in both groups (*p* = 0.19) (Table 2).

**Table 2:** Characteristic of visual analogue scale parameters, bowel movements stool consistency after consuming oleic acid

Characteristic	Distal small intestine	Stomach	*p* value
Fasting time (hours)	14.8 ± 0.5	14.8 ± 0.3	0.90
Meal consumed (g)	236.6 ± 21.7	229.8 ± 34.5	0.75
Meal consumed (kcal)	289.9 ± 28.1	281.6 ± 32.7	0.19
Sleep (hours)	6.9 ± 0.4	6.9 ± 0.3	0.89
Baseline hunger	3.5 ± 0.8	3.5 ± 1.0	0.98
Baseline fullness	4.1 ± 0.7	3.0 ± 0.7	0.26
Baseline nausea	0.6 ± 0.3	0.4 ± 0.2	0.37

Data expressed as mean and SEM

*BMI* body mass index, *g* gram, *p* probability value, *SEM* standard error of the mean

### Safety of Oleic Acid

No differences in reported side effects were observed between the two groups. Adverse events were predominantly mild, temporary and did not necessitate any interventions. Specifically, patients reported experiencing stomachache (4 participants), nausea (2 participants), burping (2 participants) and flatulence (3 participants) (Table 3).

**Table 3:** Characteristic of gastrointestinal symptoms within 24 h after consumption of oleic acid

Characteristic	Active (*N* = 14)	Placebo (*N* = 14)	*p* value
Proportion of bowel movements in 24 h	92.8% (13)	64.3% (9)	0.059
Number of bowel movements in 24h	2.5 ± 0.4	1.1 ± 0.3	0.009
Stool consistency of first stool	3.9 ± 0.5	2.4 ± 0.6	0.03
Stool consistency of stool in 24 h	4.0 ± 0.5	2.3 ± 0.5	0.03
Drink acceptance	3.8 ± 0.8	3.0 ± 0.6	0.12
Presence of urgency in first movement	21.4% (3)	14.3% (2)	0.66
Presence of urgency in 24 h	21.4% (3)	0% (0)	0.083
Presence of straining in first movement	35.7% (5)	28.6% (4)	0.41
Presence of straining in 24 h	28.6% (4)	35.7% (5)	0.65
Presence of leakage in first movement	0% (0)	0% (0)	1.00
Presence of leakage in 24 h	7.1% (1)	0% (0)	0.32
Presence of stomachache	35.7% (5)	7.1% (1)	0.10
Presence of increased burping	14.3% (2)	7.1% (1)	0.32
Presence of increased flatulence	21.4% (3)	21.4% (3)	1.00
Presence of nausea	14.3% (2)	14.3% (2)	1.00

*n* number, *H* hours, *p* probability, % percentage

## Discussion

The findings of our study indicate an improvement in constipation following a single dose of oleic acid delivered to the distal small intestine using microcapsules, with no observed safety concerns. This suggests that encapsulated oleic acid may offer benefits for patients with constipation or potentially enhance the effects of laxatives. The enhanced stimulation in the gastrointestinal tract by oleic acid could potentially improve the neural tissue regulation of autonomic sensory afferent neurons, thereby stimulating bowel movements. Delivery of oleic acid to the distal bowel might counteract sympathetic activation associated with bariatric surgery or the effects of GLP-1 analogues on increasing the ileal brake, thus potentially reducing the retardation of proximal gastrointestinal motility and inhibition of secretions seen after obesity treatments.

Another potential mechanism may involve the pro-kinetic of undigested fat to the colon, similar to the laxative properties observed with orlistat [23–25]**.** However, in our study, there were no reports of steatorrhea after the first 24 h of treatment. Delivery of oleic acid to the distal intestine may also improve peristalsis via the neural afferent intestinal signalling pathway and further change intestinal microbiota [27].

Surprisingly, we observed no difference in calorie consumption at the ad libitum meal after the administration of oleic acid microcapsules, contrary to our hypothesis and the results of a previous study [28]. This may be attributed to the fact that most of our participants were optimally treated with bariatric surgery and/or GLP-1 analogues, suggesting that these patients may have an optimal calorie intake for their respective adipocyte mass and thus adding oleic acid may not further contribute to obesity treatment. The dose of oleic acid used was also 20% lower in the present study (400 kcal) than in our previous study (500 kcal) which showed that encapsulated fat released in the distal small intestine led to enhance gut hormone responses and reduction of calorie intake [22]. Moreover, a substantial amount of the oleic acid is likely to be excreted together with the faeces because this is the mechanism how it reduces constipation. Therefore, the additional 400 kcal of olive oil delivered to the distal small intestine is unlikely to result in a calorie surplus in patients treated with bariatric surgery and/ or GLP-1 analogues.

Despite the promising results, our study has several limitations. The sample size was adequate for the research question but not large enough to be generalizable to the entire population. The single-centre design further limits the generalizability of the data but reduced potential variability between multiple sites. The single dose of the investigative material and short-term follow-up data do not allow for long-term predictions, but both the significant benefit and low side effect profile are reassuring as a long-term study is planned. Additionally, our cohort was predominantly female, reflecting the demographic of patients treated with bariatric surgery and GLP-1 analogues for obesity. Nonetheless, the treatment appeared safe, with very few patients developing diarrhoea or significant gastrointestinal symptoms. However, long-term safety profile of encapsulated oleic acid needs to be investigated in a long-term multiple-dose study such as a phase 3 trial. In light of the study results, we expect the safety profile of the microcapsules will be good because the drink provided to patients consists of natural pea protein and olive oil. Moreover, the release of the oleic acid in the distal small intestine means the additional calories are unlikely to all be absorbed but will rather soften the motions and increase peristalsis of the colon to address constipation. Longer term studies with larger numbers of patients will also be able to address whether the increased gut hormone secretion and reduced food intake observed in healthy volunteers without constipation [22] is also observed in patients after bariatric surgery with constipation.

In conclusion, this study demonstrates the feasibility of delivering microencapsulated oleic acid to the distal intestine to treat chronic constipation in patients treated for obesity with bariatric surgery and/or GLP-1 analogues, without safety concerns.

## Supplementary Information

Below is the link to the electronic supplementary material.

Supplementary file1 (DOCX 16.1 KB)

## Data Availability

Data are available on request from the corresponding author, C.L.R.
