# Supplementary material for: Laxative Properties of Microencapsulated Oleic Acid Delivered to the Distal Small Intestine in Patients with Constipation after Bariatric Surgery or Treatment with Glucagon-Like- Peptide 1 Analogues
Source: Obes Surg. 2024 Sep 5;34(10):3807–12. doi: 10.1007/s11695-024-07492-y (PMC11464573; doi:10.1007/s11695-024-07492-y)
Supplement: Supplementary file 1 — Supplementary file1 (DOCX 16.1 KB) [file 11695_2024_7492_MOESM1_ESM.docx]

**Participants Meal Preference**

| **Meal Type** | **Percentage & Number** |
| --- | --- |
| Chicken Korma | 71.42% (10) |
| Chicken Tikka Masala | 28.57% (4) |

**MEAL 1**

**Nutritional Value of Chicken Korma with Pilau rice meal**

**Nutritional information**

| **Typical Values** | **Per 100g** | **Each pack (368g)** |
| --- | --- | --- |
| Energy | 542kJ /129kcal | 1993kJ / 475kcal |
| Fat | 5.1g | 18.6g |
| Saturates | 3.0g | 11.1g |
| Carbohydrate | 14.2g | 52.4g |
| Sugars | 1.6g | 5.9g |
| Fibre | 1.4g | 5.2g |
| Protein | 6.0g | 21.9g |
| Salt | 0.36g | 1.33g |
| * Reference intake of an average adult (8400 kJ / 2000 kcal) | | |
| ** When microwaved according to instructions 400g typically weighs 368g. | | |
| When microwaved according to instructions. | | |
| Pack contains 1 servings. | | |

Abbreviations: g: gram; kcal: kilocalorie; kJ: kilojoule

**Ingredients:** Cooked Pilau Rice (40%) (Water, Basmati Rice, Garlic Purée, Ginger Purée, Spices, Salt), Chicken Breast (19%), Water, Onion, Coconut Milk (6%), Cream (**Milk**) (5%), Tomato, Yoghurt (**Milk**), Rapeseed Oil, Tomato Paste, Spices, Ginger Purée, Garlic Purée, Corn Starch, Sugar, Salt, Desiccated Coconut, Coriander, Maltodextrin, Black Pepper, **Mustard** Powder, Ginger Powder, Yeast Extract, Flavouring, Dextrose, Garlic Powder, **Celery** Powder, Colour (Plain Caramel).

### **Allergy Information:** Contains mustard, celery and milk. The allergens in this product have changed. For allergens, see ingredients in bold.

**MEAL 2**

**Chicken Tikka Masala & Pilau rice meal**

**Nutritional Information**

| **Typical Values** | **Per 100g** | **Each pack (364g)** |
| --- | --- | --- |
| Energy | 541kJ /129kcal | 1968kJ / 468kcal |
| Fat | 4.2g | 15.1g |
| Saturates | 1.8g | 6.4g |
| Carbohydrate | 15.6g | 56.9g |
| Sugars | 1.2g | 4.2g |
| Fibre | 1.2g | 4.4g |
| Protein | 6.6g | 23.9g |
| Salt | 0.41g | 1.50g |
| * Reference intake of an average adult (8400 kJ / 2000 kcal) | | |
| Pack contains 1 servings. | | |
| When microwaved according to instructions. | | |
| ** When microwaved according to instructions 400g typically weighs 364g. | | |

Abbreviations: g: gram; kcal: kilocalorie; kJ: kilojoule

### **Allergy Information:** contains mustard, celery and milk. The allergens in this product have changed., For allergens, see ingredients in bold.

**Ingredients:** Cooked Pilau Rice (40%) (Water, Basmati Rice, Garlic Purée, Ginger Purée, Spices, Salt), Chicken Breast (19%), Water, Onion, Cream (**Milk**) (5%), Tomato, Tomato Paste, Yogurt (**Milk**), Rapeseed Oil, Spices, Garlic Purée, Corn Starch, Ginger Purée, Salt, Sugar, Red Chilli, Coriander, **Mustard** Seed, Tapioca Starch, Maltodextrin, Black Pepper, Ginger Powder, Yeast Extract, **Mustard** Powder, Parsnip Powder, Dried Onion, Spirit Vinegar, Garlic Powder, **Celery** Powder, Parsley, Colour (Plain Caramel), Sunflower Oil.
